# Effects of repeated ovarian hyperstimulation on gut microbiota composition in a mouse model

**DOI:** 10.5935/1518-0557.20260054

**Published:** 2026

**Authors:** Marcelo Borges Cavalcante, Olga Goiana Martins Sampaio, Sacha Aubrey Alves Rodrigues Santos, Léo Santiago, Marina de Barros Mamede Vidal Damasceno, Larissa Cruz de Souza, Rohit Shukla, Sidharth P Mishra, Hariom Yadav, Cristiana Libardi Miranda Furtado, Adriana Rolim Campos

**Affiliations:** 1 Graduate Program in Medical Sciences, Universidade de Fortaleza (UNIFOR), Fortaleza, CE, Brazil; 2 Medical School, Universidade de Fortaleza (UNIFOR), Fortaleza, CE, Brazil; 3 Experimental Biology Center, Universidade de Fortaleza (UNIFOR), Fortaleza, CE, Brazil; 4 Graduate Program in Biotechnology - RENORBIO, Universidade Estadual do Ceará (UECE)/Universidade de Fortaleza (UNIFOR), Fortaleza, Brazil; 5 USF Center for Microbiome Research, Microbiomes Institute, University of South Florida Morsani College of Medicine, Tampa, USA; 6 Department of Neurosurgery and Brain Repair, Center of Excellence in Aging and Brain Repair, University of South Florida Morsani College of Medicine, Tampa, FL, USA; 7 Department of Internal Medicine-Digestive Diseases and Nutrition, University of South Florida Morsani College of Medicine, Tampa, FL, USA

**Keywords:** ovarian hyperstimulation, ovulation induction, biomarker, assisted reproductive technology, gut microbiome

## Abstract

**Objective:**

This study investigated whether repeated cycles of controlled ovarian hyperstimulation (COH) influ-ence the gut microbiota composition in female mice.

**Methods:**

Eight-week-old female Swiss mice (n=20; 10 per group) underwent ten weekly COH treatments with ex-ogenous gonadotropins or received saline injections as con-trols. One week after the final treatment, fecal samples were collected for gut microbiota profiling using 16S rRNA gene sequencing (V4 region amplification; primers 515F/806R) on the Illumina MiSeq platform. Sequencing data were pro-cessed in QIIME2 with DADA2 for denoising and Green-genes-based taxonomic assignment. Alpha-diversity (Shan-non, Simpson, observed OTUs, Faith’s PD) and beta-diversity (Bray-Curtis, Jaccard, unweighted/weighted UniFrac) metrics were calculated. Differential taxonomic abundance and bio-marker analyses were performed using Random Forest and LEfSe. Statistical significance was set at *p*<0.05.

**Results:**

No significant differences were observed between COH and control groups for alpha-diversity in-dices (all *p*>0.05) or beta-diversity distances (PERMANO-VA *p*>0.05). Relative abundances across major taxonomic levels were similar, although the *Firmicutes:Bacteroidetes* ratio was lower in COH-treated mice. Biomarker analyses identified *Bacteroidales, Erysipelotrichaceae,* and *Parabac-teroides* as enriched in the COH group, while *Rikenellaceae* and *Odoribacter* were more abundant in controls.

**Conclusion:**

Repeated COH did not significantly alter overall gut microbial diversity in mice under the conditions tested. However, specific taxonomic shifts and biomark-er signatures were detected, indicating associations-not causality-between COH exposure and changes in microbi-ota composition. Further studies are needed to determine the functional and physiological implications of these as-sociations.

## INTRODUCTION

Infertility affects millions of couples worldwide and is considered a major public health concern. Its increasing prevalence is attributed to factors, such as delayed childbearing, modern lifestyles, and adverse environmental conditions (Vander Borght & Wyns, 2018). Assisted reproductive technology (ART), including *in vitro* fertilization (IVF) and intracytoplasmic sperm injection (ICSI), constitute a pivotal strategy in managing infertility. A central step in optimizing oocyte production and increasing the success rates of these treatments is controlled ovarian hyperstimulation (COH) (Ovarian Stimulation TEGGO *et al.*, 2020). Despite advances in reproductive medicine, the success rates for a single IVF cycle remain modest, ranging from approximately 30% to 50% in women under 35 years of age, with a significant decline in older age groups (Gleicher *et al.*, 2019; Sunkara *et al.*, 2023). Multiple IVF attempts are often required due to the inherent limitations of this technique. Other factors, such as the woman’s age, embryo quality, and underlying reproductive health conditions, also contribute to this requirement (Sunkara *et al.*, 2023). Thus, repeated IVF cycles are often necessary to maximize the chances of successful conception, particularly in patients facing infertility-related challenges or poor prognosis.

Briefly, COH protocols use urinary or recombinant follicle-stimulating hormone and luteinizing hormone to stimulate the development of multiple ovarian follicles, aiming to maximize the number of mature eggs obtained for subsequent fertilization and embryo transfer (Ovarian Stimulation TEGGO *et al*., 2020). As a result, while undergoing treatment with these drugs, serum estradiol concentrations reach supraphysiological levels, increasing from 150 to 400 pg/mL in a physiological menstrual cycle to between 1,000 and 6,000 pg/mL in a COH cycle, depending on the number of follicles recruited (Siddhartha *et al.*, 2016; Helmer *et al.*, 2022). The short- and long-term systemic effects of repeated COH are not yet fully elucidated, and they continue to evoke significant scientific interest. This is particularly true regarding the interactions between sex hormones and emotional, immunological, and metabolic abnormalities (Miyamoto *et al.*, 2010; Marschalek *et al.*, 2019; Hu *et al.*, 2021; Ma *et al.*, 2021; Guo *et al.*, 2023; Sampaio *et al.*, 2024). Sex steroids, particularly estrogen, play a crucial role in female reproductive physiology and interact intimately with the gut microbiota. Growing evidence suggests that fluctuations in estrogen levels can alter the composition and functionality of the gut microbiota (Yang *et al.*, 2022; Schieren *et al.*, 2024). Additionally, the gut microbiota plays a crucial role in regulating systemic estrogen levels by mediating its metabolism through the gut-liver axis (Baker *et al.*, 2017; Siddiqui *et al.*, 2022). These bidirectional interactions not only affect hormonal balance but may also influence susceptibility to immune disorders, metabolic alterations, and estrogen-dependent gynecological diseases, such as endometriosis, polycystic ovary syndrome, and endometrial cancer (Siddiqui *et al.*, 2022).

Animal models can be used to investigate the impact of estrogens on the gut microbiota. Mice and rats are among the most frequently studied species, often subjected to surgical induction (ovariectomized [OVX]) or chemical induction (treatment with 4-vinyl cyclohexene diepoxide [VCD]) of a hypoestrogenic state to evaluate the effects of hormonal supplementation on the gut microbiota (Song *et al.*, 2020). Transgenic mice are also employed to explore the specific role of estrogen receptors (ERα, ERβ) (Cook *et al.*, 2014). The zebrafish model (*Danio rerio*) offers an innovative alternative, with studies using estradiol exposure to investigate *in vivo* intestinal changes (Cornuault *et al.*, 2022). Non-human primates are typically reserved for more complex research due to their physiological similarity to humans, despite ethical and financial limitations (Yan *et al.*, 2022). Rabbit models are utilized in studies examining the interaction between estrogens and intestinal fermentation (Ericsson, 2019). These models are evaluated using techniques, such as 16S rRNA sequencing and metabolomics, to elucidate the estrogen’s effects on microbiota diversity and composition, particularly through bile acid and secondary metabolite modulation (Guo *et al.*, 2023).

Repeated COH, which clinically refers to multiple cycles of COH used in ART, has not been directly associated with alterations in gut microbiota composition or diversity in humans. Despite the established significance of the gut microbiota-hormone axis, research on the impact of repeated COH protocols on the gut microbiota remains limited. It is imperative to evaluate how these interventions may affect gut microbiota and potentially influence pregnancy outcomes. Additionally, estrogen and its fluctuations are vital for both overall and reproductive health, making it essential to understand their role in the onset and progression of diseases in both the short- and long-term. The present study aimed to investigate whether COH induces alterations in the gut microbiota composition of female mice. Given the known interactions between sex hormones and microbial communities, we hypothesized that supraphysiological estrogen fluctuations resulting from repeated COH protocols could modulate gut microbial diversity and structure. By employing a well-established animal model subjected to weekly COH for ten consecutive weeks, this study seeks to provide novel insights into the potential impact of sustained hormonal stimulation on gut microbiota dynamics, contributing to a better understanding of the systemic effects of ART.

## MATERIAL AND METHODS

### Animals

For this study, we used 8-week-old sexually mature female Swiss mice, weighing between 20 and 30 g. The animals were sourced from the Vivarium at the Experimental Biology Center, University of Fortaleza. A total of 20 animals were used, and they were randomly assigned to two experimental groups (n=10 per group)-control and treatment-using a computer-generated randomization sequence to ensure unbiased allocation. The animals were housed in individually ventilated cages (Techniplast IVC), with autoclaved pine shavings used as bedding. The mice were maintained under pathogen-free conditions, with an average ambient temperature of 26°C, 15-20 air changes per hour, and a 12-hour light/ dark cycle. They were provided *ad libitum* access to filtered water and commercial chow, both sterilized prior to use to maintain microbiological safety. All experimental procedures followed international guidelines for the care and use of laboratory animals and were approved by the Animal Use Ethics Committee of the University of Fortaleza (CEUA protocol number 3828200123). Furthermore, this study adhered to the principles outlined in the Animal Research: Reporting of In Vivo Experiments (ARRIVE 2.0) guidelines, to ensure transparency and reproducibility in animal research (Percie du Sert *et al.*, 2020).

### Repeated ovarian hyperstimulation

**Figure 1:**
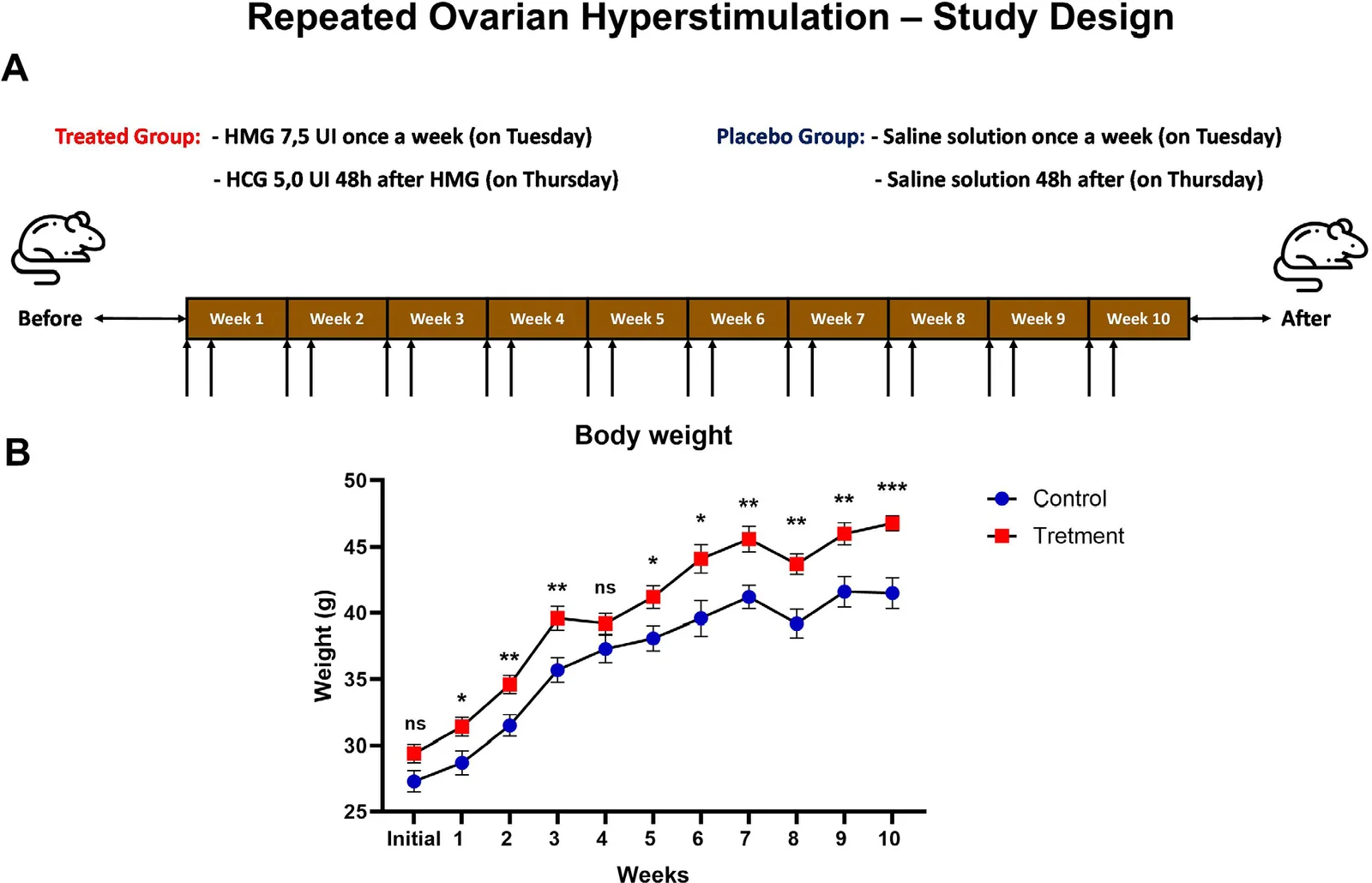
Effects of Repeated Ovarian Hyperstimulation on Body Weight in Female Mice. (A) Experimental design of repeated ovarian hyperstimulation in female mice. The treated group received 7.5 IU of human menopausal gonadotropin (HMG) once per week (on Tuesdays) followed by 5.0 IU of human chorionic gonadotropin (hCG) 48 hours later (on Thursdays) for 10 weeks. The placebo group received saline solution at equivalent time points. (B) Body weight progression of treated and control groups during the 10-week protocol. Data are presented as mean±SEM, with significant differences between groups marked as follows: *p<0.05, **p<0.01, ***p<0.001, and ns for not significant. Treated mice exhibited a significant increase in body weight compared to controls starting from the second week.

The mice (n=10 per group) were randomly assigned to one of the two experimental groups: placebo (control) or treatment. The treatment group underwent ovarian hyperstimulation following established protocols described in the literature (Van Blerkom & Davis, 2001; Zhang *et al.*, 2018). Specifically, mice in the treatment group received an intraperitoneal (i.p.) injection of 7.5 IU of human menopausal gonadotropin (HMG, Menopur™, FERRING Pharmaceuticals), a gonadotropin preparation containing both FSH and LH, to stimulate follicular development. After 48 hours, mice received a second i.p. injection of 5 IU of human chorionic gonadotropin (hCG, Choriomon™, IBSA Institut Biochimique), a hormone that mimics the LH surge and induces ovulation. In contrast, mice in the control group were administered an equivalent volume of saline solution (i.p.) at the same time points. Ovulation was induced ten times, with intervals of one week between each induction (Figure 1A).

### Physical characteristics, 16S rRNA gene sequenc-ing, and analysis of feces

Mice were weighed one week prior to the beginning of the experimental protocol, weekly throughout the ten-week COH treatment, and again one week after the final administration of either ovulation-inducing agents or saline placebo. Fecal samples were collected from each animal one week after the conclusion of the treatment phase and were immediately frozen at -80°C for subsequent analysis. Genomic DNA was extracted from approximately 100 mg of murine fecal material using the QIAamp® PowerFecal® Pro DNA kit (Qiagen, CA, USA), following the manufacturer’s instructions. The composition of the gut microbiota was assessed using 16S rRNA gene sequencing on the Illumina MiSeq platform, employing bioinformatics methodologies as previously described (Nagpal *et al.*, 2020a;b; Mishra *et al.*, 2023). The V4 hypervariable region of the bacterial 16S rDNA gene was amplified using universal primer pairs 515F (5’-GTGCCAGCMGCCGCGGTAA-3’) and 806R (5’-GGACTACHVGGGTWTCTAAT-3’) (Caporaso *et al.*, 2012). Barcoded amplicons were then purified with AMPure® magnetic beads (Agencourt, Beckman Coulter, CA, USA), quantified using the dsDNA HS assay kit (Life Technologies, Carlsbad, CA, USA) on a Qubit-3 fluorometer (Invitrogen, Carlsbad, CA, USA), and normalized for constructing an amplicon library (Caporaso *et al.*, 2012). Each amplicon was standardized to a final concentration of 8 pM before sequencing on the Illumina MiSeq system (MiSeq reagent kit v3).

We then used the QIIME2 (Quantitative Insights into Microbial Ecology) software to process the generated fastq files from the sequencing. The quality control, clustering, and demultiplexing of the sequences were performed using the unique barcodes assigned to each sample. Subsequently, quality control evaluations were conducted using the DADA2 pipeline (Nagpal *et al.*, 2019; 2020b; Mishra *et al.*, 2024). High-quality sequences were obtained by removing adapters and non-chimeric amplicons using the default parameters of DADA2. The filtered sequences were then used for the taxonomy classification using the Greengenes-trained Naive Bayes classifier (Bokulich *et al.*, 2018).

Alpha-diversity indices for community richness included the observed operational taxonomic units (OTUs), Shannon index, Simpson index, and Faith’s phylogenetic diversity index. Community dissimilarities (beta diversity) were quantitatively assessed using unweighted UniFrac, weighted UniFrac, Jaccard index, and Bray-Curtis distances within QIIME2, and these were visualized with a principal coordinate analysis (PCoA) plot. To obtain relative abundances, raw read counts were divided by the total number of reads per sample. Subsequently, OTUs were aggregated to taxonomic levels by summing their respective relative abundances.

### Statistics

GraphPad Prism 10.2.0 software (GraphPad Software Inc., La Jolla, CA, USA) was used for statistical analysis. Body weight was described as the mean and standard error of the mean (mean±SEM). For analysis of the alpha diversity indices (Observed OTUs, Shannon, Simpson, and Faith’s Phylogenetic Diversity), comparisons between the control and COH-treated groups were conducted using a two-tailed unpaired Student’s *t*-test, assuming equal variance unless otherwise specified. Statistical significance was defined as *p*<0.05. To identify differentially abundant taxa between groups, LEfSe (Linear Discriminant Analysis Effect Size) was applied, using default parameters with an LDA score threshold>2.0 and a significance level of *p*<0.01, as previously described by Segata *et al*. (2011). In addition, a Random Forest classification algorithm was used to evaluate taxa importance and identify microbial features that best distinguished between the two experimental groups. Beta diversity distances (Jaccard, Bray-Curtis, unweighted and weighted UniFrac) were statistically compared using PERMANOVA (permutational multivariate analysis of variance) within the QIIME2 framework.

## RESULTS

Body weights were collected weekly from both experimental groups: control (n=10) and treatment (n=10). Longitudinal analysis of weight change revealed that both groups experienced progressive weight gain. However, the treated group showed significantly greater weight gain starting from the fourth week of COH (Figure 1B).

**Figure 2:**
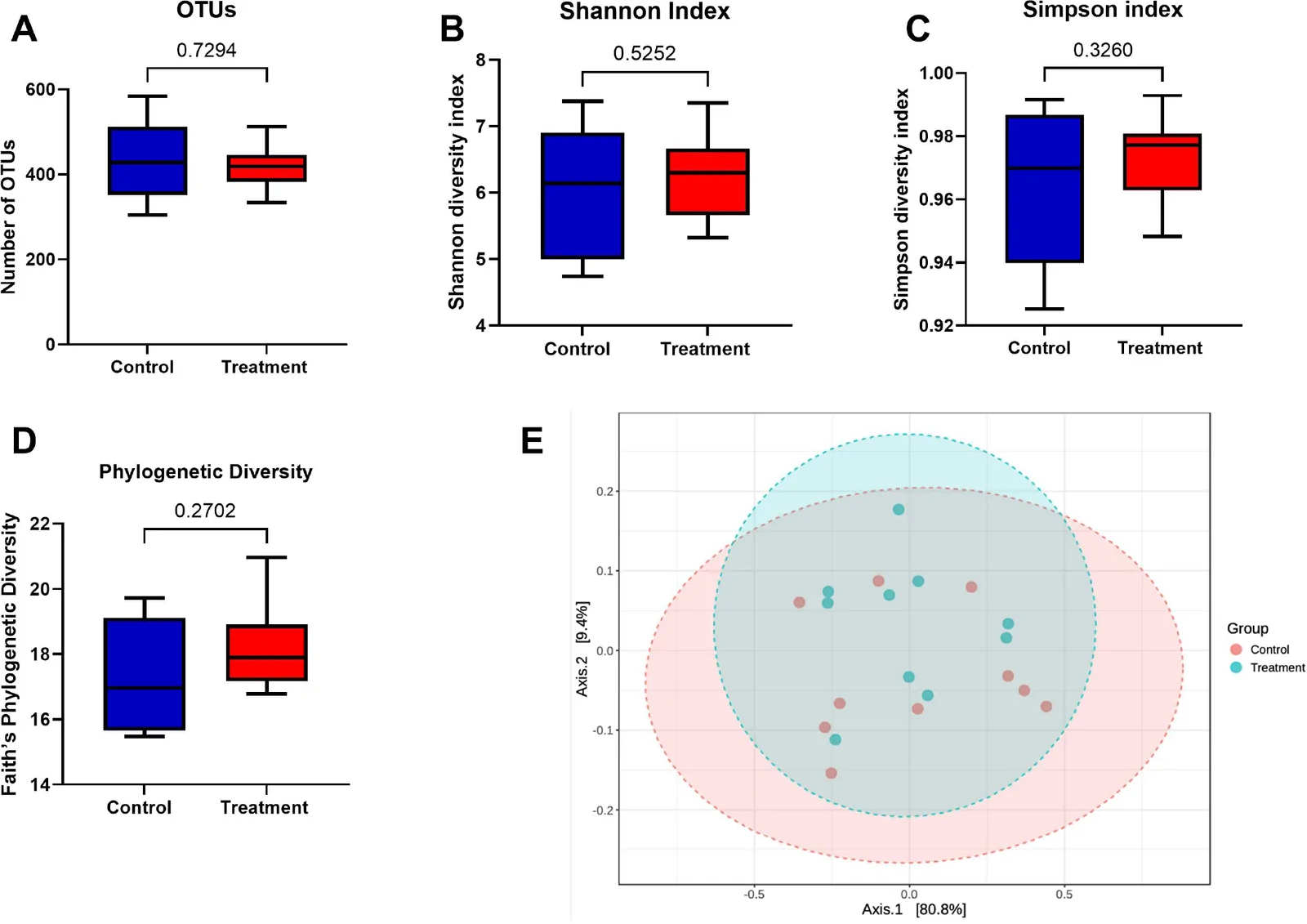
Impact of Repeated Controlled Ovarian Hyperstimulation (COH) on Alpha and Beta Diversity of the Gut Microbiota in Female Mice. Female Swiss mice (n=10 per group) were assigned to a treatment group receiving repeated COH for 10 weeks or a placebo-treated control group. (A-D) Alpha diversity was assessed using four metrics: (A) Observed OTUs (Control: 430.5±29.7; Treatment: 418.7±15.6; *p*=0.7294), (B) Shannon diversity index (Control: 6.02±0.98; Treatment: 6.27±0.67; *p*=0.5252), (C) Simpson diversity index (Control: 0.96±0.02; Treatment: 0.97±0.01; *p*=0.3260), and (D) Faith’s Phylogenetic Diversity (Control: 17.41±1.73; Treatment: 18.19±1.29; *p*=0.2702). Data are presented as mean±standard deviation (SD). No significant differences were observed between groups for any of the alpha diversity metrics (unpaired two-tailed t-test). (E) Beta diversity was evaluated using weighted UniFrac distances, and visualized via principal coordinates analysis (PCoA). Each dot represents a fecal sample; ellipses denote 95% confidence intervals for each group. No clear clustering by group was observed (PERMANOVA; *p*>0.05).

The results of the alpha and beta diversity analyses are summarized in Figure 2. The boxplots compare the alpha-diversity metrics between the control (blue) and treatment (red) groups. The alpha diversity of the samples was assessed by counting the number of OTUs and calculating the Shannon, Simpson, and Faith’s Phylogenetic Diversity (PD) indices. Overall, the findings suggest that the repeated COH protocol did not significantly affect the richness or PD of the microbial community in the studied groups (Figures 2A-D). There was no significant difference in species richness, as measured by observed OTUs, between the control group (430.5±93.8) and the treatment group (418.7±45.3) (mean±SD; *p*=0.7294, η^2^=0.0068) (Figure 2A). The Shannon diversity index, which accounts for both species richness and evenness, did not differ significantly between the treatment group (6.27±0.67) and the control group (6.02±0.98) (mean±SD; *p*=0.5252, η^2^=0.0227), indicating similarly high levels of microbial diversity in both groups (Figure 2B). The Simpson diversity index, which reflects species dominance within the microbial community, did not differ significantly between the treatment group (0.97±0.01) and the control group (0.96±0.02) (mean±SD; *p*=0.3260, η^2^=0.0536). The high Simpson values observed in both groups indicate a community structure characterized by strong dominance of a few taxa, consistent with low overall evenness (Figure 2C). Faith’s PD did not differ significantly between the treatment group (18.19±1.29) and the control group (17.41±1.73) (mean±SD; *p*=0.2702, η^2^=0.0670) (Figure 2D), indicating comparable levels of evolutionary diversity across the microbial communities in both groups.

Beta diversity analysis was conducted using the weighted UniFrac index, which accounts for the phylogenetic distance between microbial communities, weighted by the relative abundance of species. A PCoA plot revealed overlapping ellipses for the control and treatment groups, indicating no substantial differences in the overall composition of microbial communities. The first principal axis (PC1) accounted for 80.8% of the total variation, while the second principal axis (PC2) contributed 9.4%. Beta diversity analysis did not reveal distinct clusters between groups, indicating no major changes in the microbial pattern due to treatment (Figure 2E). However, this may be attributed to the smaller sample size.

**Figure 3:**
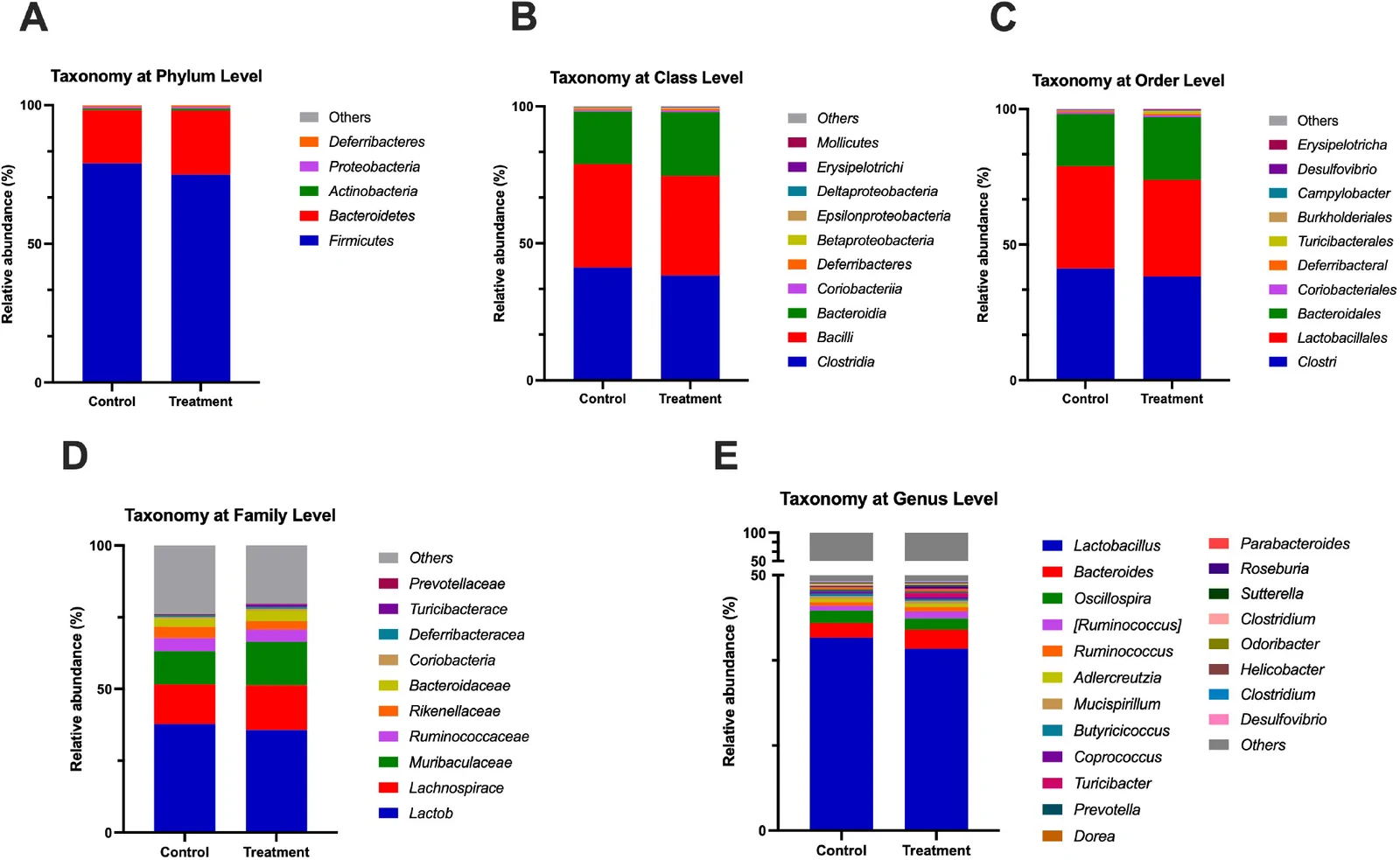
Taxonomic Composition of Gut Microbiota at Multiple Levels Following Repeated Controlled Ovarian Hyperstimulation (COH) in Female Mice. This figure presents the relative abundance (%) of bacterial taxa at the phylum (A), class (B), order (C), family (D), and genus (E) levels in control mice (n=10) and mice subjected to 10 weeks of COH (n=10). At the phylum level (A), *Firmicutes* and *Bacteroidetes* dominated both groups, with a modest reduction in the Firmicutes:Bacteroidetes ratio in the treatment group. At the class level (B), *Clostridia* and *Bacteroidia* remained predominant, while minor increases in *Erysipelotrichi* and *Coriobacteriia* were observed after COH. At the order level (C), *Bacteroidales* showed a slight increase in treated mice, whereas *Clostridiales* were more abundant in controls. At the family level (D), *Lactobacillaceae, Lachnospiraceae*, and *Ruminococcaceae* were the major families across groups, with *Rikenellaceae* showing a small decrease in the COH group. At the genus level (E), *Lactobacillus* was the dominant genus in both groups, followed by *Bacteroides* and *Parabacteroides*, which were slightly more abundant in treated animals, while genera such as *Rikenellaceae_RC9* and *Clostridium* were relatively enriched in controls. These patterns indicate largely conserved community structure, with subtle COH-associated shifts in specific taxa.

The gut microbiota’s taxonomic composition was evaluated across five levels: phylum, class, order, family, and genus. Phylum-level analysis revealed that the treatment group had a higher relative abundance of *Bacteroidetes* (23.1% in the treated group *vs*. 19.2% in the control group) and a lower percentage of *Firmicutes* (74.9% in the treated group *vs*. 78.9% in the control group) as compared to the control group (Figure 3A). The *Firmicutes: Bacteroidetes* (F: B) ratio was reduced by the COH protocol, decreasing to 3.24 in the treatment group compared to 4.11 in the control group. *Proteobacteria, Actinobacteria*, and *Deferribacteres* constituted a minor fraction of the total community and maintained consistent proportions across both groups (Figure 3A). The gut microbiota composition at the class level was primarily dominated by *Clostridia, Bacilli,* and *Bacteroidia.* We observed a reduction in *Clostridia* and *Bacilli*, while *Bacteroidia* was elevated in the treatment group compared to the control group. Minor classes, including *Coriobacteriia, Deferribacteres, Epsilonproteobacteria, Deltaproteobacteria, Betaproteobacteria, Erysipelotrichi,* and *Mollicutes*, showed no significant differences between the groups, indicating that the COH protocol did not notably alter class-level microbial distributions (Figure 3B).

At the order level, the control group exhibited an increase in abundance of *Clostridiales* and *Lactobacillales*, while the treatment group exhibited a higher abundance of *Bacteroidales*. Other orders, including *Coriobacteriales, Deferribacterales, Burkholderiales, Campylobacterales, Turicibacterales, Desulfovibrionales*, and *Erysipelotrichales*, showed comparable relative abundances, indicating that the treatment protocol did not substantially affect these microbial taxa (Figure 3C). At the family level, the *Lactobacillaceae, Ruminococcaceae,* and *Rikenellaceae* showed increased abundance in the control group, while the treatment group exhibited higher abundance for *Lachnospiraceae, Muribaculaceae,* and *Bacteroidaceae* (Figure 3D). At the genus level, *Lactobacillus* and *Oscillospira* were more abundant in the control group, while *Bacteroides, Ruminococcus, Turicibacter*, and *Parabacteroides* were more abundant in the treatment group (Figure 3E).

**Figure 4:**
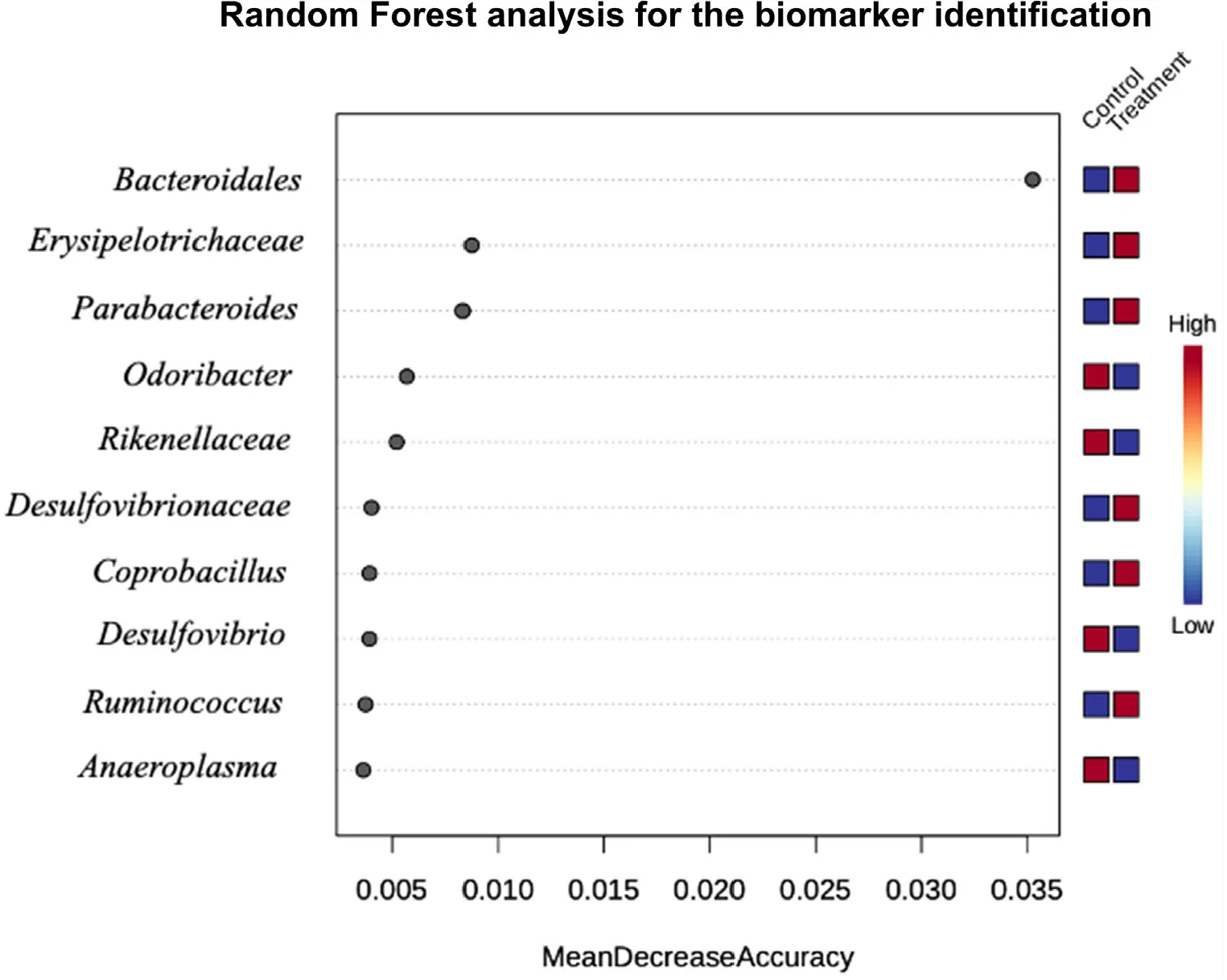
Random Forest Analysis Identifies Key Microbial Biomarkers Associated with Controlled Ovarian Hyperstimulation (COH). Random forest classification was performed to identify bacterial taxa that most accurately distinguish fecal microbiota profiles of COH-treated mice (n=10) from control mice (n=10). The x-axis shows the Mean Decrease Accuracy, reflecting the contribution of each taxon to model performance. The y-axis lists the most discriminative taxa, with *Bacteroidales, Erysipelotrichaceae*, and *Parabacteroides* emerging as top predictors. The heatmap on the right illustrates the relative abundance of each taxon across groups, with the color scale ranging from red (high abundance) to blue (low abundance). Notably, *Bacteroidales* was more abundant in the COH group and ranked as the most informative biomarker, suggesting its relevance in microbial shifts associated with ovarian hyperstimulation.

Two taxa were statistically significant: *Mogibacteriaceae* (*p*=0.049) and *Clostridiaceae* (*p*=0.038) at the family level. At the genus level, four genera were significant: *Parabacteroides* (*p*=0.034), *Anaeroplasma* (*p*=0.041), *Candidatus Arthromitus* (*p*=0.029), and *Clostridium* (*p*=0.031). Furthermore, the random forest analysis indicated that *Bacteroidales* was the most abundant in the treatment group, serving as a discriminating biomarker between the two groups. We also observed that one bacterial family, *Erysipelotrichaceae*, and three genera, *Parabacteroides, Odoribacter*, and *Rikenellaceae*, could serve as biomarkers. *Erysipelotrichaceae* and *Parabacteroides* were more abundant in the treatment group, while *Odoribacter* and *Rikenellaceae* were more abundant in the control group (Figure 4).

## DISCUSSION

This study investigated whether repeated COH, a condition that raises endogenous estrogen concentrations to supraphysiological levels, can induce changes in the gut microbiota of female mice. The biological plausibility for the link between hormonal fluctuations (estrogen and progesterone) and microbial changes is supported by data showing that estrogen promotes proliferation of the vaginal epithelium and increased glycogen, favoring the growth of *Lactobacillus*, while progesterone modulates glycogen release and vaginal pH. Furthermore, hormonal variations throughout the menstrual cycle and in states such as hyperestrogenism induced by controlled ovarian stimulation alter the diversity and composition of the vaginal microbiota, with *Lactobacillus* predominating during periods of higher estrogen levels (Collins *et al*., 2022).

The results of this study revealed that prolonged COH treatment in female mice does not significantly affect the diversity (alpha or beta) of the gut microbiota, as assessed by the metrics presented. The results also suggested that the 10-week COH protocol does not induce notable changes in the taxonomic structure of the gut microbiota in female mice. The stability observed at several taxonomic levels reinforces the notion that the COH protocol has a limited impact on the microbial community structure. However, the study identified biomarkers of gut microbiota, including one order, one family, and three genera. The most distinguishing factor between the groups was the abundance of bacteria from the order *Bacteroidales* in the group subjected to repeated COH. Furthermore, the presence of one family (*Rikenellaceae*) and two genera (*Parabacterioides* and *Odoribacter*) within the order *Bacteriodales* was identified as potential biomarkers of gut microbiota. The only biomarker not belonging to the order *Bacteroidales* was the family *Erysipelotrichaceae*.

Alterations in the gut microbiota were observed at different phases of the menstrual cycle and during the menopausal transition in healthy women. These findings suggest that fluctuations in physiological levels of endogenous estrogen significantly influence the composition of the gut microbiota (Krog *et al.*, 2022; Schieren *et al.*, 2024). Higher estrogen levels were associated with a higher abundance of *Bacteroidetes*, a lower abundance of *Firmicutes* and the *Ruminococcaceae* family, as well as greater diversity (Zhao *et al.*, 2019; Krog *et al.*, 2022; Schieren *et al.*, 2024). Studies in animal models have demonstrated that estrogen supplementation can significantly influence the diversity and composition of the gut microbiota (Kaliannan *et al.*, 2018; Acharya *et al.*, 2019; Song *et al.*, 2020). However, studies are limited to estrogen supplementation via oral gavage, i.p. injection, or subcutaneous implants, with no studies evaluating supraphysiological fluctuations of endogenous estrogen.

Song *et al*. (2020) observed that 7β-estradiol (E2, 10 mg/kg, i.p. injection) increased alpha diversity, as measured by the number of OTUs observed, in male mice. Interestingly, alpha diversity, measured by the Shannon index, increased in female mice after OVX, a condition that induces low levels of endogenous estrogens. The authors also observed that estrogen levels influenced the composition of the gut microbiota across all taxonomic levels. At the phylum level, the abundance ratio of *Cyanobacteria* significantly decreased in male mice receiving E2, whereas the abundance ratio of *Verrucomicrobia* significantly increased in the absence of estrogen (OVX). E2 supplementation or ovariectomy did not affect the F: B ratio. In male mice, E2 supplementation increased the commensal bacteria/ opportunistic pathogens ratio (control male=1.5 *vs*. E2 male=5) (Song *et al.*, 2020). Acharya *et al*. (2019) also reported that E2 supplementation (via subcutaneous implants) in female OVX mice alters the gut microbial composition, altering alpha diversity (observed number of OTUs) and beta diversity (Bray-Curtis dissimilarity). This supplementation reduced the relative abundance of *Firmicutes* and *Actinobacteria* while increasing the presence of *Bacteroidetes* (Acharya *et al.*, 2019).

Studies have shown that menopause is associated with significant changes in the composition of the gut microbiota, probably influenced by estrogen levels (Baker *et al.*, 2017; Yang *et al.*, 2022; Huang *et al.*, 2024). Yang *et al*. (2022) observed a higher relative abundance of the genera *Odoribacter* and *Bilophila* in postmenopausal women. In contrast, Zhao *et al.* (2019) identified a depletion of *Firmicutes* and *Roseburia* spp., along with an overrepresentation of *Bacteroidetes* and *Tolumonas* in the same population (Zhao *et al.*, 2019; Yang *et al.*, 2022). Santos-Marcos *et al.* (2018) corroborated these findings, reporting a higher F: B ratio and increased abundance of *Lachnospira* and *Roseburia* in premenopausal women, highlighting the influence of hormonal status on the microbiota (Santos-Marcos *et al.*, 2018). In animal models, Dai *et al*. (2023) identified microbial and metabolic changes during the menopausal transition, associated with neuroendocrine aging. However, there is a gap in understanding microbiota changes at earlier stages, such as premenarche and menarche.

The interaction between estrogen levels and the gut microbiota is complex, involving both the modulation of microbial composition by estrogens and the influence of the microbiota on estrogen metabolism through the estrobolome (Baker *et al.*, 2017; Siddiqui *et al.*, 2022; Huang *et al.*, 2024). Studies suggest that estrogen may indirectly modulate the microbiota through interactions with the immune system and bile, both of which play a role in selecting microorganisms in the intestinal tract (Salliss *et al.*, 2021; Huang *et al.*, 2024). Many bacterial genera and species in the intestine contain genes encoding β-glucuronidase and β-galactosidase (Markowitz *et al.*, 2012). Our results observed that *Parabacteroides* and *Odoribacter*, genera expressing β-galactosidase and indirectly involved in the estrobolome, were markers of gut microbiota composition.

The changes in the gut microbiota observed in our study differed from those reported in other studies, especially because they did not affect alpha or beta diversity. However, most studies published previously have examined the effect of E2 supplementation on the gut microbiota composition in animal models of menopause, comparing a low estrogen condition with sex hormone replacement. Our study compared animals with normal estrogen levels to a group experiencing supraphysiological estrogen fluctuations for ten consecutive weeks. This study is pioneering in exploring the effects of repeated COH on gut microbiota composition, providing important insights into the interplay between reproductive interventions and gut health. A key strength of this study is the identification of a potential link between supraphysiological estrogen levels and changes in the gut microbiota, a novel finding that underscores the systemic effects of hormonal manipulation. By highlighting estrogen’s role in modulating gut microbial composition, the study opens new avenues for understanding how reproductive technologies, such as ovarian hyperstimulation, may have broader implications for women’s health. These implications are particularly relevant in terms of metabolism, immunity, and overall well-being.

Changes in the composition of the intestinal microbiota observed in our study may have significant implications for reproductive health through immune and metabolic pathways. *Bacteroidales* are known to enhance the production of short-chain fatty acids (SCFAs), which play a crucial role in maintaining intestinal integrity and modulating systemic inflammation. Their ability to influence cytokine production and reduce lipopolysaccharide levels may contribute to a lower systemic inflammatory state, which is particularly relevant in reproductive disorders where chronic inflammation can impair implantation and pregnancy maintenance (Fabersani *et al.*, 2021). *Erysipelotrichaceae*, associated with reduced intestinal inflammation, may further support reproductive health by promoting a balanced immune environment, potentially decreasing the risk of immune-mediated pregnancy complications such as implantation failure or recurrent pregnancy loss (Zhuang *et al*., 2022). Similarly, *Parabacteroides* have been linked to anti-inflammatory and metabolic benefits, improving glucose homeostasis and reducing obesity-related inflammation-factors that are critical in conditions such as polycystic ovary syndrome and infertility associated with metabolic dysfunction (Cui *et al*., 2022).

The decrease of *Rikenellaceae* and *Odoribacter* in the gut microbiota may also have significant immune and metabolic implications. These bacterial genera are important producers of SCFAs, particularly butyrate, which serves as an energy source for colonocytes and has anti-inflammatory properties that regulate both intestinal and systemic immune responses (Zarrinpar *et al*., 2018; Amabebe *et al*., 2020). A reduction in *Rikenellaceae* may lead to a decrease in butyrate production, compromising intestinal barrier integrity, increasing permeability and promoting bacterial translocation and endotoxemia, which are associated with chronic low-grade inflammation, insulin resistance and obesity (Zarrinpar *et al*., 2018; Amabebe *et al*., 2020). Similarly, reduction of *Odoribacter* further decreases SCFA production and disrupts metabolic and immune homeostasis (Zarrinpar *et al*., 2018; Amabebe *et al*., 2020). Overall, the loss of these bacterial taxa may contribute to gut inflammation, metabolic dysregulation and impaired immune function, highlighting their critical role in maintaining gut health.

The pattern of changes in gut microbiota composition in our study may promote beneficial or detrimental effects on gut health and may have implications for reproductive health. Further research is needed to clarify the long-term consequences, considering that host-microbiota interactions are influenced by multiple factors, such as diet, hormonal fluctuations, and individual metabolic profiles.

Despite these strengths, the study has limitations that affect the interpretation of its results. Most importantly, the absence of estrogen monitoring throughout the protocol prevents a detailed understanding of the relationship between hormonal fluctuations and microbial changes. In addition, the study does not identify the specific timing of microbiota changes during the 10-week ovarian stimulation protocol. Additionally, the study is limited by its single timepoint analysis of fecal samples and absence of functional data (e.g., metagenomics or host immune markers). This leaves a gap in our understanding of when and how these changes occur. These weaknesses limit the ability to establish causality and the precise mechanisms underlying the observed microbiota shifts. They highlight the need for future studies with more comprehensive temporal and hormonal monitoring to better elucidate these interactions.

Future research should aim to elucidate the long-term effects of repeated COH on gut microbiota composition and its potential systemic and reproductive implications. A key area of investigation is determining whether the observed microbial shifts persist beyond the treatment period or if the microbiota gradually returns to its baseline state. Additionally, exploring the role of probiotics supplementation prior to COH presents an exciting avenue for mitigating potential microbial disturbances. Probiotics have been shown to influence gut microbiota composition, immune regulation, and metabolic homeostasis, all of which are relevant to reproductive health. Future studies should evaluate whether probiotic interventions can prevent or attenuate COH-induced microbial alterations and, in turn, improve metabolic and reproductive outcomes. These investigations would not only enhance our understanding of the gut-reproductive axis but also contribute to optimizing strategies in ART to promote better clinical outcomes.

## CONCLUSION

This study investigated the impact of repeated COH on the gut microbiota in female mice. The results demonstrated that COH did not significantly alter microbial diversity (alpha and beta diversity); however, it induced notable changes in the taxonomic composition of the gut microbiota. Specifically, COH was associated with a reduced F: B ratio and an increased abundance of microbial taxa. Biomarker analyses further identified potential discriminatory taxa, including *Bacteroidales, Erysipelotrichaceae, Parabacteroides, Odoribacter*, and *Rikenellaceae*, as markers of microbial shifts in response to COH. These findings highlight the potential systemic implications of repeated COH exposure on gut microbial composition, which may contribute to broader physiological changes associated with hormonal modulation. The study emphasizes the need for further research to investigate the long-term effects of COH on gut health, immune regulation, and metabolic outcomes, particularly in the context of ART. By elucidating these interactions, future studies could help develop strategies to mitigate the unintended consequences of COH on overall health.

## Abbreviations

ART Assisted reproductive technology

COH Controlled ovarian hyperstimulation

dsDNA Double-stranded DNA

ER Estrogen receptors

hCG Human chorionic gonadotropin

HMG Human menopausal gonadotropin

ICSI Intracytoplasmic sperm injection

IP Intraperitoneal

IVF *in vitro* fertilization

LDA Linear discriminatory analysis

LEfSe LDA Effect Size

OTUs Operational taxonomic units

OVX Ovariectomized

PCoA Principal coordinate analysis

PD Phylogenetic Diversity

SEM Standard error of the mean

VCD 4-vinyl cyclohexene diepoxide
